# Peanut Shell-Derived Carbon Solid Acid with Large Surface Area and Its Application for the Catalytic Hydrolysis of Cyclohexyl Acetate

**DOI:** 10.3390/ma9100833

**Published:** 2016-10-15

**Authors:** Wei Xue, Lijun Sun, Fang Yang, Zhimiao Wang, Fang Li

**Affiliations:** Hebei Provincial Key Laboratory of Green Chemistry & High Efficient Energy Saving, School of Chemical Engineering and Technology, Hebei University of Technology, Tianjin 300130, China; weixue@hebut.edu.cn (W.X.); greenchem309@gmail.com (L.S.); fyang_greenchem@yeah.net (F.Y.); wzmcat309@gmail.com (Z.W.)

**Keywords:** carbon solid acid, peanut shell, large surface area, cyclohexyl acetate, hydrolysis

## Abstract

A carbon solid acid with large surface area (CSALA) was prepared by partial carbonization of H_3_PO_4_ pre-treated peanut shells followed by sulfonation with concentrated H_2_SO_4_. The structure and acidity of CSALA were characterized by N_2_ adsorption–desorption, scanning electron microscopy (SEM), X-ray powder diffraction (XRD), ^13^C cross polarization (CP)/magic angle spinning (MAS) nuclear magnetic resonance (NMR), X-ray photoelectron spectroscopy (XPS), Fourier transform-infrared spectroscopy (FT-IR), titration, and elemental analysis. The results demonstrated that the CSALA was an amorphous carbon material with a surface area of 387.4 m^2^/g. SO_3_H groups formed on the surface with a density of 0.46 mmol/g, with 1.11 mmol/g of COOH and 0.39 mmol/g of phenolic OH. Densities of the latter two groups were notably greater than those observed on a carbon solid acid (CSA) with a surface area of 10.1 m^2^/g. The CSALA catalyst showed better performance than the CSA for the hydrolysis of cyclohexyl acetate to cyclohexanol. Under optimal reaction conditions, cyclohexyl acetate conversion was 86.6% with 97.3% selectivity for cyclohexanol, while the results were 25.0% and 99.4%, respectively, catalyzed by CSA. The high activity of the CSALA could be attributed to its high density of COOH and large surface area. Moreover, the CSALA showed good reusability. Its catalytic activity decreased slightly during the first two cycles due to the leaching of polycyclic aromatic hydrocarbon-containing SO_3_H groups, and then remained constant during following uses.

## 1. Introduction

Cyclohexanol is an important intermediate in the chemical fiber industry for the production of nylon-6 and nylon-66 [1]. The industrial production of cyclohexanol relies mainly on the oxidation of cyclohexane [2], which has certain drawbacks, such as high energy consumption, a large amount of by-products, and the danger of explosion. In the 1990s, Asahi Chemical Industry Co. developed a process to synthesize cyclohexanol via the hydration of cyclohexene [3], which is a promising route because of very high selectivity and minimal disposal problems, owing to the use of an acidic zeolite catalyst, HZSM-5. However, this reaction is restricted by the poor miscibility of water and cyclohexene, and the reaction rate is very low. The yield from one cycle of Asahi’s process is only ~10%, therefore requiring multiple cycles with high energy consumption.

**Scheme 1:**
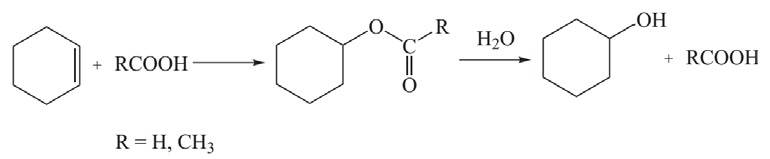
Synthesis of cyclohexanol from cyclohexene via cyclohexyl carboxylate.

Fortunately, the synthesis of cyclohexanol from cyclohexene via cyclohexyl carboxylate—which can be referred to as an indirect hydration of cyclohexene—overcomes the above-mentioned drawbacks in the direct hydration of cyclohexene [4]. It is a two-step process, comprising the esterification of cyclohexene with carboxylic acid and the following hydrolysis of the formed cyclohexyl carboxylate to cyclohexanol (Scheme 1).

**Scheme 2:**
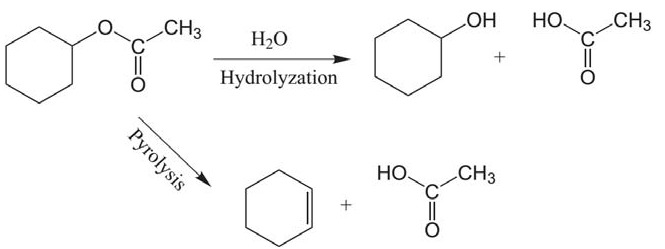
Cyclohexyl acetate hydrolysis and pyrolysis reactions.

Steyer and Sundmacher studied the indirect hydration of cyclohexene via cyclohexyl formate over Amberlyst-15 in a reactive distillation column [5,6]. They concluded that it is possible to achieve almost complete conversion of cyclohexene to cyclohexanol. In our previous work, we studied a one-pot synthesis of cyclohexanol from cyclohexene via cyclohexyl formate over HZSM-5 [7]. Cyclohexanol was formed in yields of up to 40%, which is considerably higher than those obtained by direct hydration of cyclohexene. The reaction between cyclohexene and acetic acid, and the subsequent hydrolysis of cyclohexyl acetate over HZSM-5 was also studied [8]. It was found that cyclohexene conversion for the first step was 69.1% with 98.3% cyclohexyl acetate selectivity, which was almost the same as that seen in the reaction between cyclohexene and formic acid. However, there was a large difference between the hydrolysis of cyclohexyl acetate and that of cyclohexyl formate. At 130 °C, 63.4% of cyclohexyl acetate was converted over HZSM-5, with only 26.9% cyclohexanol selectivity. Most of the cyclohexyl acetate underwent pyrolysis to cyclohexene. Scheme 2 shows these reaction routes.

Several groups have used the hydrolysis of cyclohexyl acetate as a probe reaction to test catalytic materials [9,10,11]. In their results, pyrolysis of cyclohexyl acetate was not mentioned, and cyclohexanol was the sole product. This may be attributable to the low reaction temperature (80 °C) used in their studies, inhibiting the pyrolysis reaction. In our previous work [12], we found that the ionic liquid (IL) 1-sulfobutyl-3-methyl-imidazolium hydrogen sulfate ((HSO_3_bmim)HSO_4_) could be used as a catalyst to increase the selectivity of cyclohexanol in the hydrolysis of cyclohexyl acetate. Under optimal conditions, the cyclohexyl acetate conversion was 80.2%, with 96.6% cyclohexanol selectivity at 130 °C. These excellent results may be attributable to the high polarity of the ILs, which inhibits the formation of the transition state for the side reaction, pyrolysis of cyclohexyl acetate to cyclohexene, and then increases cyclohexanol selectivity. However, the high cost and complexity of the production of the ILs limits their application.

In recent years, carbon solid acids (CSAs) have been studied by many researchers [10,13,14,15,16,17,18,19,20,21,22,23,24,25]. A CSA is an amorphous carbon material consisting of small polycyclic aromatic carbon sheets with attached SO_3_H groups [10]. Because of their high densities of SO_3_H groups, simple separation process, and environmentally friendly characteristics, CSAs are considered to be promising replacements for H_2_SO_4_, which is one of the most widely used homogeneous acid catalysts for the production of many industrially important chemicals [14]. Hara et al. [10] prepared a CSA catalyst by the sulfonation of naphthalene in concentrated H_2_SO_4_ that exhibited higher activity for the hydrolysis of cyclohexyl acetate than did H_2_SO_4_. However, they reported no detailed results from their experiments. Moreover, the CSA they used had a surface area of only 24 m^2^/g—in most cases, the specific surface area of CSA is less than 10 m^2^/g. Kitano et al. [26] indicated that a CSA exhibited high catalytic performance in the presence of hydrophilic molecules despite its relatively low specific surface area (2 m^2^/g), which they attributed to the incorporation of high densities of hydrophilic molecules into the carbon bulk by binding with the flexible carbon sheets. However, because the hydrophilic functional groups also prevent the incorporation of hydrophobic molecules into the bulk, acid-catalyzed hydrophobic reactions can only proceed on the catalyst surface, resulting in poor or no catalytic activity for such reactions. Therefore, a strong solid acid catalyst with high specific surface area is more desirable for acid-catalyzed hydrophobic reactions.

In this paper, a carbon solid acid with large specific surface area (CSALA) was prepared by carbonization of H_3_PO_4_-impregnated peanut shell powder followed by sulfonation. The catalytic performance of this carbon catalyst for the hydrolysis of cyclohexyl acetate to cyclohexanol was investigated. The results exhibited that the CSALA showed much higher activity than that of the CSA with small surface area. The cause is discussed and its reusability is also explored.

## 2. Results and Discussion

### 2.1. Catalyst Characterization

The surface areas of the CSA, CSALA, and their precursors were investigated by N_2_ adsorption–desorption at −196 °C. As shown in Table 1, the specific surface area of the CSALA is 387.4 m^2^/g, which is much higher than that of the CSA. The results show that H_3_PO_4_ is an effective activating agent, which can lead to the formation of porosity in the activated carbon from lignocellulosic materials [27].

**Figure 1:**
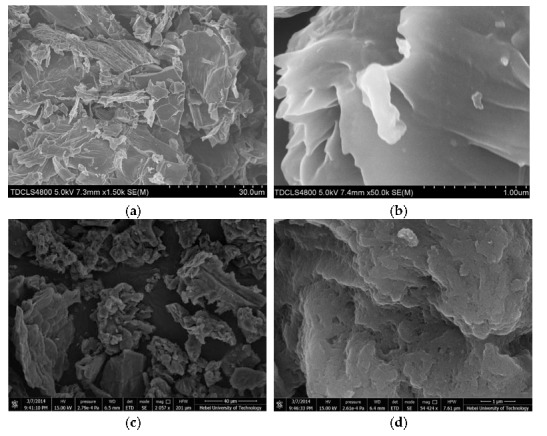
Scanning electron microscopy (SEM) images of CSA (**a**,**b**) and CSALA (**c**,**d**).

Scanning electron microscopy (SEM) images, shown in Figure 1, also illustrate the differences between the surface morphologies of the CSA and CSALA. Figure 1a,b show SEM images of the CSA, which exhibits a lamellar structure that is composed of polycyclic aromatic carbon sheets. The CSALA has a similar lamellar structure, but the sheets stack much more loosely. The CSALA carbon sheets are also smaller and have rougher surfaces than the CSA sheets. All of these differences are responsible for the porosity and increased specific surface area of the CSALA compared with the CSA.

**Figure 2:**
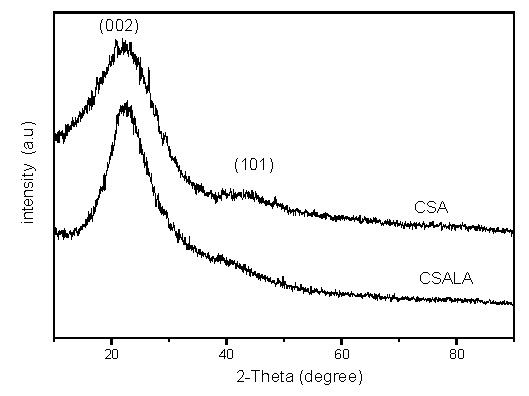
X-ray powder diffraction (XRD) patterns of CSA and CSALA.

Figure 2 shows the XRD patterns of the CSA and CSALA. In both patterns, there is one broad diffraction peak at 2θ angles of 20°–30° (002), which reflects the stacking degree of aromatic carbon sheets [28], and one weak peak at 40°–50° (101), which is attributable to the α axis of the graphite structure [14]. The XRD results indicate that both the CSA and CSALA consist of disordered polycyclic aromatic carbon sheets. However, compared with that of the CSA, the (101) peak of the CSALA is almost non-existent. This corresponds to the smaller carbon sheets in the CSALA, and is consistent with the SEM results.

**Figure 3:**
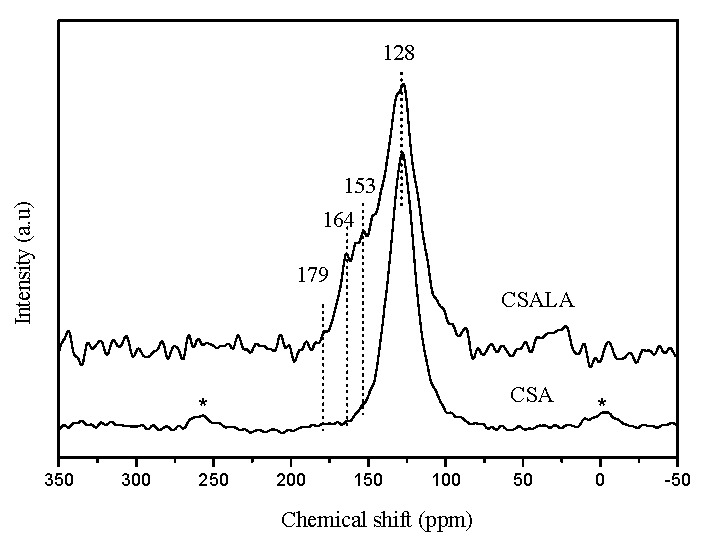
^13^C CP/magic angle spinning (MAS) NMR spectra of CSA and CSALA. * denotes spinning sidebands.

**Figure 4:**
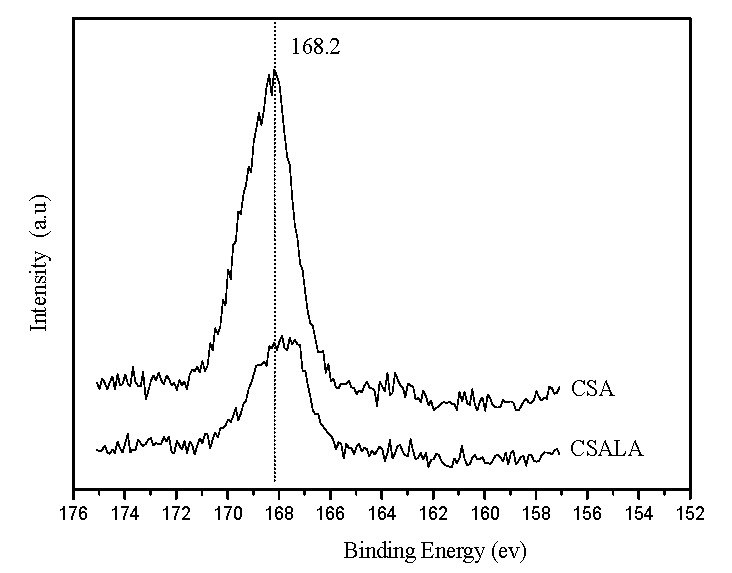
S 2p X-ray photoelectron spectroscopy (XPS) spectra of CSA and CSALA.

Figure 3 shows the ^13^C CP/magic angle spinning (MAS) NMR spectra of the CSA and CSALA. The signals at 128 ppm attributable to polycyclic aromatic carbon atoms are evident in both spectra, indicating that the carbon frameworks of these two materials consist mainly of sp^2^-derived carbon sheets. Peaks attributable to aromatic carbon bonded to phenolic OH (153 ppm), COOH groups (164 ppm), and carbon atoms in COOH groups (179 ppm) are clearly observed in the spectrum of CSALA. However, the corresponding signals in the CSA spectrum are very weak. This demonstrates the difference between the amounts of phenolic OH and COOH groups in the CSA and CSALA. Moreover, the peaks attributable to aromatic carbon with SO_3_H groups (ca. 140 ppm) [29] are not obvious in either the CSA or CSALA spectra. This may be attributable to the polycyclic aromatic carbon peak (128 ppm) obscuring the peak of Ar-SO_3_H. The introduction of SO_3_H groups to the carbon solid acids was confirmed by X-ray photoelectron spectroscopy (XPS) analysis. As shown in Figure 4, a single S 2p peak at about 168 eV—which is assigned to the SO_3_H group—is observed in both spectra. This indicates that the sulfonation of carbon materials was successfully achieved in concentrated H_2_SO_4_, and all S atoms in the CSA and CSALA are contained in SO_3_H groups [20].

**Figure 5:**
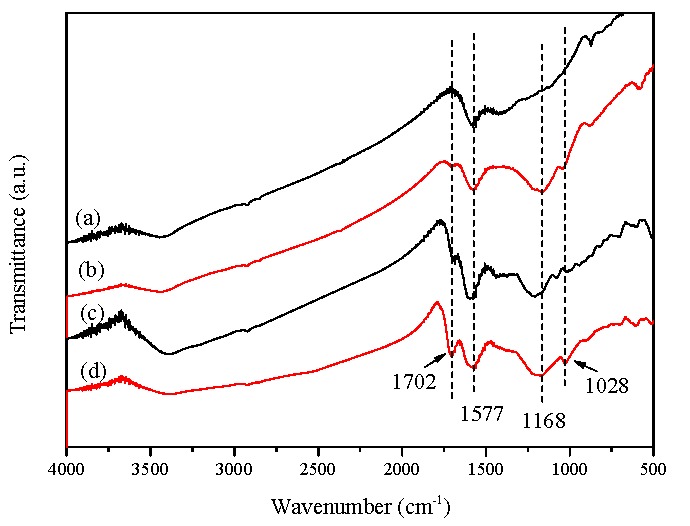
Fourier transform-infrared (FT-IR) spectra of carbon materials: (**a**) pre-CSA; (**b**) CSA; (**c**) pre-CSALA; (**d**) CSALA.

Fourier transform-infrared spectroscopic (FT-IR) analysis was used to characterize the functional groups contained in the carbon materials. As shown in Figure 5, there are two bands at 1168 cm^−1^ and 1028 cm^−1^ in the CSALA spectrum, which can be assigned to the O=S=O symmetric and asymmetric stretching modes, respectively [30]. The same bands also appear in the spectrum of CSA. This illustrates that sulfonic acid groups have been formed on the surface of the CSA and CSALA by the sulfonation process. The band at 1702 cm^−1^ is attributable to the C=O stretching mode of the COOH groups. There is also a clear adsorption attributable to C=O in the COOH group in the spectrum of pre-CSALA. The generation of COOH groups can be attributed to the treatment with phosphoric acid. Moreover, the evident band around 1577 cm^−1^ seen in all the spectra is attributable to the C=C stretching vibration of poly aromatic rings [31].

The acid densities of the carbon materials were determined by acid–base titration, and the results are given in Table 1. The total acid density of the CSALA is 1.96 mmol/g, which is higher than that of the CSA (1.12 mmol/g). However, the density of SO_3_H in the CSALA (0.46 mmol/g) is less than that in the CSA (0.56 mmol/g); further, the main contribution to acid density in the CSALA comes from COOH (1.11 mmol/g), which is far greater than in the CSA (0.44 mmol/g). In addition, the density of COOH in pre-CSALA is 1.08 mmol/g, which is almost the same as that in the carbon material after sulfonation. This agrees reasonably well with the FT-IR results. The abundant COOH in pre-CSALA could partially explain why the CSALA has less SO_3_H than the CSA. The sulfonation of polycyclic aromatic compounds is an electrophilic substitution reaction; therefore, the electron-withdrawing COOH could decrease the electron density of the aromatic ring, and thus deactivate the sulfonation reaction. This would lead to fewer SO_3_H groups in the CSALA, even though it has a larger surface area than the CSA. Finally, the density of phenolic OH in the CSALA (0.39 mmol/g) is also much greater than that in the CSA (0.12 mmol/g).

### 2.2. Hydrolysis of Cyclohexyl Acetate over the CSALA

Catalytic hydrolysis of cyclohexyl acetate was evaluated, and the results are shown in Table 1. The CSALA exhibits good catalytic performance, and cyclohexyl acetate conversion was 81.1%, with 95.7% cyclohexanol selectivity. However, the CSA—with higher SO_3_H content—shows poor activity for this reaction (25.0% cyclohexyl acetate conversion with 99.4% cyclohexanol selectivity). This indicates that SO_3_H is not the only active site, and there must be other factors influencing the reaction. Because there are many COOH groups in the CSALA, it is reasonable to speculate that COOH can also catalyze this reaction. To confirm this conjecture, pre-CSALA (which has almost the same COOH content as CSALA) was evaluated for the hydrolysis of cyclohexyl acetate. It was found that cyclohexyl acetate conversion could reach 31.4% with 97.9% cyclohexanol selectivity, even though there are no SO_3_H groups in pre-CSALA. The results indicate that COOH can also catalyze the hydrolysis of cyclohexyl acetate. However, the total conversion over pre-CSALA and CSA catalysts is still inferior to that over CSALA. This may be attributable to the high specific surface area of CSALA, which can provide good access for the hydrophobic cyclohexyl acetate molecules to approach the active acid sites [26]. Therefore, a higher cyclohexyl acetate conversion was obtained under the synergistic effect of a high COOH density and the large specific surface area of CSALA.

#### 2.2.1. Effect of Reaction Temperature

**Figure 6:**
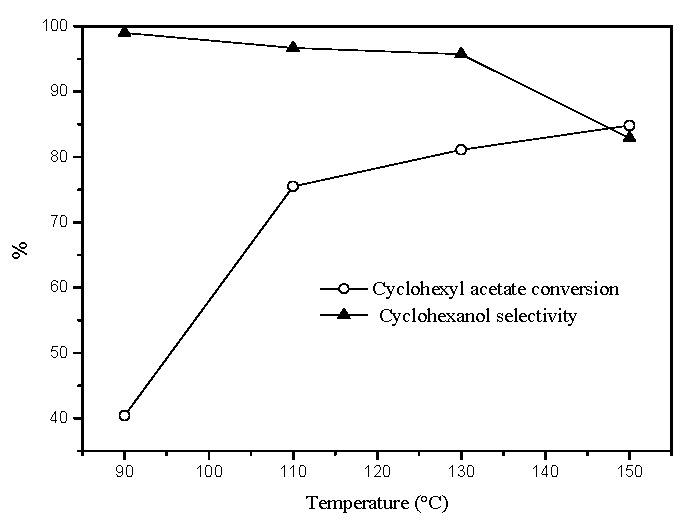
Effect of reaction temperature on the hydrolysis of cyclohexyl acetate over CSALA. Reaction conditions: cyclohexyl acetate 8 mL (55 mmol), H_2_O 24 mL (1333 mmol), CSALA 1.2 g, 5 h.

Figure 6 shows the effect of the reaction temperature on the hydrolysis of cyclohexyl acetate over CSALA catalyst. It can be seen that cyclohexyl acetate conversion increased rapidly from 40.4% to 75.5% with the reaction temperature increasing from 90 to 110 °C. With further increases of the reaction temperature, cyclohexyl acetate conversion slightly increased and reached 84.8% at 150 °C. However, selectivity to cyclohexanol decreased gradually from 99.0% to 95.9% with the temperature increasing from 90 to 130 °C. After that, it decreased rapidly to 82.8% at 150 °C, and the main side product was cyclohexene. This indicates that the pyrolysis of cyclohexyl acetate occurred to a greater extent at high temperatures.

#### 2.2.2. Effect of Reaction Time

**Figure 7:**
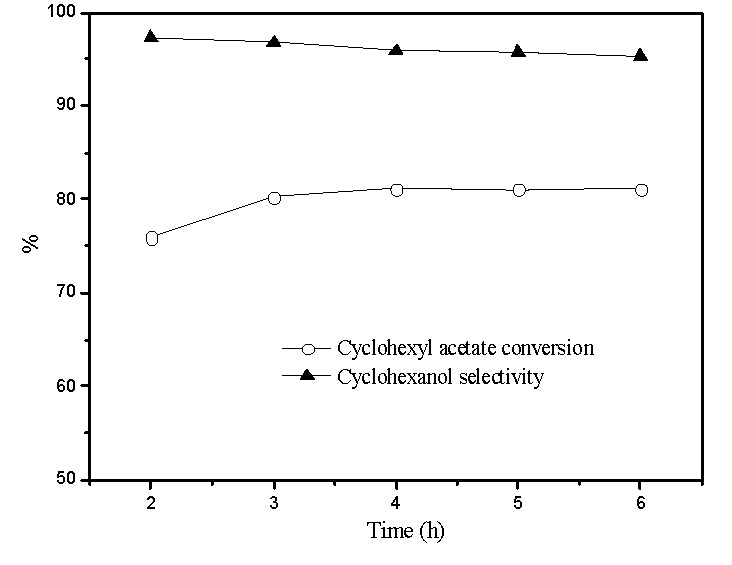
Effect of reaction time on the hydrolysis of cyclohexyl acetate over CSALA. Reaction conditions: cyclohexyl acetate 8 mL (55 mmol), H_2_O 24 mL (1333 mmol), CSALA 1.2 g, 130 °C.

Figure 7 shows the effect of reaction time on the hydrolysis of cyclohexyl acetate over the CSALA. When the reaction time was prolonged from 2 to 3 h, cyclohexyl acetate conversion increased from 75.9% to 80.3%. With a further increase of reaction time, conversion reached a plateau. Moreover, cyclohexanol selectivity declined slightly from 97.3% to 95.9% when the reaction time increased from 2 to 4 h, after which it remained unchanged. This indicates that cyclohexyl acetate is prone to undergoing pyrolysis to cyclohexene during prolonged reaction times, but the change was not notable.

#### 2.2.3. Effect of Catalyst Loading

**Figure 8:**
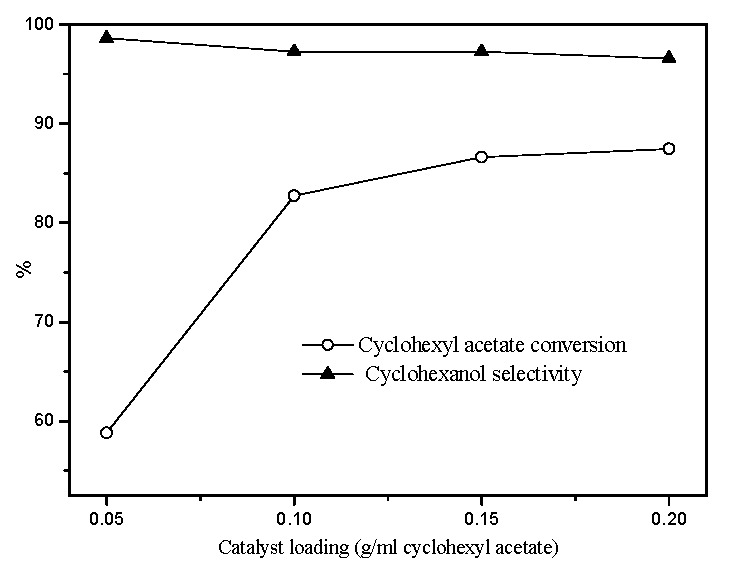
Effect of catalyst loading on the hydrolysis of cyclohexyl acetate over the CSALA. Reaction conditions: cyclohexyl acetate 8 mL (55 mmol), H_2_O 40 mL (2222 mmol), 130 °C, 3 h.

The effect of CSALA catalyst loading on the hydrolysis of cyclohexyl acetate was investigated, and the results are shown in Figure 8. Cyclohexyl acetate conversion initially increased, and then reached a plateau with increasing CSALA loading. When the catalyst loading was 0.15 g CSALA/mL cyclohexyl acetate, the conversion reached 86.6% with 97.3% cyclohexanol selectivity. However, the selectivity to cyclohexanol showed a slight monotonic decrease from 98.8% to 96.6% in the catalyst loading range investigated. This indicates that the pyrolysis of cyclohexyl acetate can also be accelerated by the CSALA and is more sensitive to catalyst loading. Under the same stirring conditions, too much catalyst would result in a low mass transfer coefficient; thus, a low reaction rate for the hydrolysis was obtained. However, pyrolysis of cyclohexyl acetate is a monomolecular reaction and is not greatly affected by diffusion.

#### 2.2.4. Catalyst Reusability

**Figure 9:**
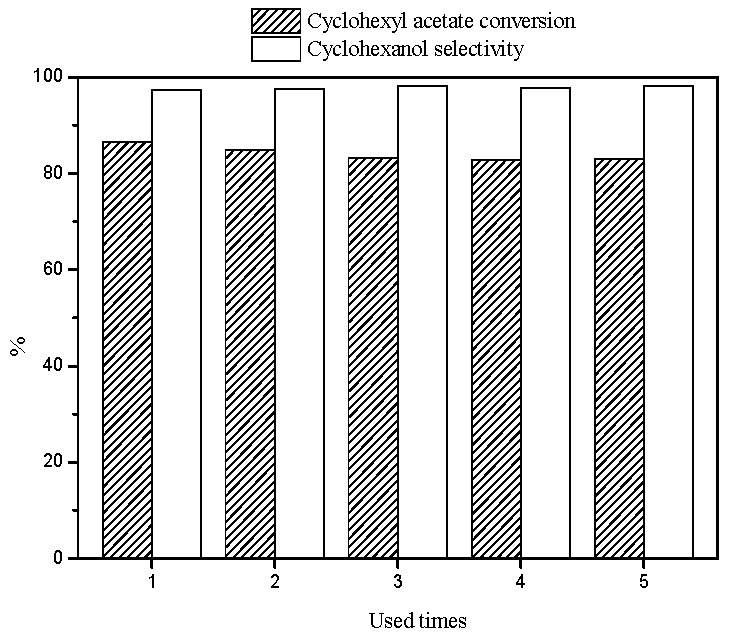
Reusability of CSALA for the hydrolysis of cyclohexyl acetate. Reaction conditions: cyclohexyl acetate 8 mL (55 mmol), H_2_O 40 mL (2222 mmol), CSALA 1.2 g, 130 °C, 3 h.

The reusability of the CSALA for the hydrolysis of cyclohexyl acetate was investigated. After the first run, the catalyst was filtered from the mixture, washed with water, and dried. It was then used directly in a new cycle without any further treatment. As shown in Figure 9, high selectivity (not less than 97.3%) for cyclohexanol was obtained in every cycle; however, the cyclohexyl acetate conversion decreased from 86.6% to 84.9% (second cycle) to 83.2% (third cycle). After this, the conversion remained constant with reuse from the third to the fifth cycles. The CSALA catalyst that had been used for five cycles was characterized by elemental analysis, and the S content was 1.51 wt %. Compared with the fresh CSALA S content of 1.86 wt %, it can be concluded that the activity decrease was partly caused by S (SO_3_H) leaching. Although sulfonated carbons are considered to be insoluble in some solvents [14], some researchers have found that the catalytic deactivation of carbon solid acids was caused by the leaching of polycyclic aromatic hydrocarbon containing SO_3_H groups, especially in polar media [32]. The catalytic activity of CSALA decreased during the initial cycles, but reached a plateau after three cycles in this study. This indicated that the leaching of the active species on the CSALA does stop. This may be attributable to the different natures of the acid sites—some sites are readily leached, while others are not. It has been estimated that around 75%–80% of acid sites in a sulfonated carbon catalyst are relatively stable and are not readily leached [32]. This is in accordance with the elemental analysis results. In addition, it can be seen that the activity loss is not proportional to the decrease of the S content. This may be explained by the fact that SO_3_H groups are not the only active sites. As mentioned above, both the high density of COOH and the high surface area of CSALA contribute greatly to the high activity.

## 3. Materials and Methods

### 3.1. Preparation of Carbon Solid Acid with Large Surface Area

The CSALA was prepared using peanut shells as the raw material. First, the peanut shells were dried overnight at 80 °C. They were then ground into particles of 0.15–0.2 mm (80–100 mesh). The particles were then impregnated in a phosphoric acid solution (1.22 g/mL) at room temperature for 16 h, at a 3:1 mass ratio of phosphoric acid solution-to-peanut shell. After drying at 80 °C, the impregnated peanut shells were calcined at 300 °C under N_2_ flow for 2 h. After calcination, the sample was washed with HCl solution (0.1 mol/L) at 100 °C for 1 h and then washed repeatedly with hot deionized water (≥80 °C) until free of Cl^−^. After this, the sample was dried at 110 °C to obtain the carbon precursor for the CSALA (denoted as pre-CSALA). In the next step, the pre-CSALA was sulfonated with concentrated H_2_SO_4_ (25 mL/g pre-CSALA) at 160 °C for 10 h under N_2_ flow. Finally, the solid carbon material was washed with hot deionized water (≥80 °C) until the filtrate was free from SO_4_^2−^ and dried at 80 °C to obtain the CSALA.

For comparison, a CSA with a small surface area was also prepared. First, peanut shells were dried overnight at 80 °C. They were then ground into particles of 1–5 mm. After that, the peanut shells were calcined at 550 °C for 10 h under a N_2_ flow to obtain the carbon precursor for the CSA (denoted as the pre-CSA). Next, the pre-CSA was sulfonated with concentrated H_2_SO_4_ (25 mL/g pre-CSA) at 160 °C for 10 h under N_2_ flow. Finally, the solid carbon material was washed with hot deionized water (≥80 °C) until the filtrate was free from SO_4_^2−^ and dried at 80 °C to obtain the CSA. 

### 3.2. Catalyst Characterization

The specific surface area and pore structure of the samples were measured by N_2_ isothermal adsorption–desorption method using a Micromeritics ASAP 2020 instrument. The surface area was calculated by Brunauer–Emmett–Teller (BET) method. The morphologies of the samples were recorded by scanning electron microscopy (SEM) on FEI Nova NanoSEM 450 (Hillsboro, OR, USA) and Hitachi S-4800 scanning electron microscopes (Tokyo, Japan). The X-ray diffraction (XRD) pattern was observed on a Rigaku D/Max-2500 X-ray diffractometer (Tokyo, Japan) with Cu Kα radiation operating at 40 kV and 100 mA, over a 2θ range of 5°–80° at a scanning speed of 8°/min. ^13^C CP/MAS NMR spectra were measured at room temperature using a Bruker AV 600 spectrometer (MAS probe: 4 mm and spectra window: 75 kHz, Karlsruhe, Germany). The magic angle spinning rate was 4.5 kHz. FT-IR spectra were obtained on a Nicolet Nexus 470 spectrometer (Madison, WI, USA) with KBr pellet technique. X-ray photoelectron spectroscopy (XPS) was carried out with a Thermo Fisher K-Alpha spectrometer (Tewksbury, MA, USA) with Al Kα radiation. The composition of the samples was determined by the elemental analysis of C, H, N, S, and O using an Elementar Vario EL elemental analyzer (Frankfurt, Germany).

The acid density of the catalyst was estimated by acid–base titration method. To obtain SO_3_H density, about 0.25 g of catalyst sample was put into a NaCl solution (0.05 mol/L, 30 mL). Then, the mixture was sonicated for 1 h in an ultrasonic bath at room temperature. After that, the solids were filtered off and washed with water. The combined filtrate was titrated with a NaOH solution (0.05 mol/L) using phenolphthalein as indicator. The densities of SO_3_H were thus obtained based on the results.

The densities of other acidic functional groups of the carbon solid acid—such as carboxyl and phenolic hydroxyl—can be determined by Boehm titration method [33]. About 0.5 g of catalyst was sonicated with 25 mL 0.05 mol/L NaOH (or NaHCO_3_) solution for 1 h and then soaked for 24 h. After filtration, 50 mL of 0.05 mol/L HCl was added into the solution. Then, the excess HCl were measured by anti-titration with NaOH (0.05 mol/L) using phenolphthalein as indicator. The acid densities—SO_3_H + COOH + OH (or SO_3_H + COOH)—could be calculated with the results. At last, the quantities of COOH and OH can be obtained by subtracting the density of SO_3_H.

### 3.3. Catalytic Activity Test

Hydrolysis of cyclohexyl acetate was carried out in a stainless steel Teflon-lined autoclave equipped with a magnetic stirrer. In a typical experiment, cyclohexyl acetate, H_2_O, and an appropriate amount of catalyst were added into the autoclave. The autoclave was sealed and tested for leaks with N_2_, then purged and pressurized with N_2_ (0.6 MPa). The temperature was adjusted to the desired value. At the end of reaction, the autoclave was cooled to room temperature and vented. The catalyst was recovered by filtration, and the products were analyzed by capillary gas chromatography using an Agilent 7890 instrument (Santa Clara, CA, USA) with a 30 m × 0.32 mm KB-Wax column and flame ionization detector (FID). The area normalization was applied for quantitative analysis.

## 4. Conclusions

A peanut shell-derived carbon solid acid with large specific surface area (CSALA, 387.4 m^2^/g) exhibits high activity for the hydrolysis of cyclohexyl acetate to cyclohexanol. At the optimal reaction conditions (reaction for 3 h at 130 °C; H_2_O-to-cyclohexyl acetate volume ratio of 5:1; and catalyst loading of 0.15 g/mL of cyclohexyl acetate), the cyclohexyl acetate conversion is 86.6% with 97.3% selectivity for cyclohexanol. Compared with a CSA with small surface area (10.1 m^2^/g), the CSALA catalyst shows much better activity. This is because of the high density of COOH, which is also active to hydrolysis of cyclohexyl acetate, as well as the large surface area, which can provide good access for the hydrophobic cyclohexyl acetate molecules to approach the active acid sites. The initial slight decreases of the catalytic activity during the first two reuses are due to the leaching of polycyclic aromatic hydrocarbon containing SO_3_H groups. Because of the different natures of the acid sites in the catalyst, the leaching stops, and the catalytic activity remains stable in the succeeding reusability tests.

## Figures and Tables

**Table 1:** Catalytic performance of carbon solid acid (CSA) for the hydrolysis of cyclohexyl acetate.

Catalyst	S_BET_^a^ (m^2^/g)	Acid Density (mmol/g)				Cyclohexyl Acetate Conversion (%)	Cyclohexanol Selectivity (%)
		SO_3_H	COOH	OH	Total		
pre-CSA	11.0	–	0.01	0.01	0.02	1.6	81.0
CSA	10.1	0.56	0.44	0.12	1.12	25.0	99.4
Pre-CSALA	977.3	–	1.08	0.06	1.14	31.4	97.9
CSALA	387.4	0.46	1.11	0.39	1.96	81.1	95.7
HZSM-5 ^b^	416.1	–	–	–	1.70 ^c^	63.4	26.9

Reaction conditions: cyclohexyl acetate 8 mL (55 mmol), H_2_O 24 mL (1333 mmol), catalyst 1.2 g, 130 °C, 5 h. ^a^ BET specific surface area; ^b^ Reproduced with permission from Yang et al, Reaction Kinetics, Mechanisms and Catalysis; published by Springer, 2016; ^c^ Measured by NH_3_-TPD. CSALA: carbon solid acid with large specific surface area.
